# Notes on Hæmoptysis, with Illustrative Cases.—I

**Published:** 1909-02-13

**Authors:** 


### Medicine.

### NOTES ON HÆMOPTYSIS, WITH ILLUSTRATIVE CASES.—I.

The term hæmoptysis should include blood derived from bronchus, trachea, or larynx, but not from the fauces, nares, pharynx, or mouth. When a patient brings up blood, the first consideration must be to determine whether it is a case of hæmoptysis or of hæmatemesis. These two conditions can sometimes be distinguished with ease, sometimes not at all, and sometimes only with more or less difficulty. It is necessary to inquire carefully into the history of the symptoms, to examine the blood that has been brought up, and to make as gentle a physical examination as possible, avoiding percussion of the chest for the time being and disturbing the patient as little as may be.

The following case illustrates the difficulty that may arise in distinguishing hæmoptysis from hæmatemesis.

A man, aged thirty-eight, was admitted to hospital on January 1 for bringing up blood. His father had died of gout, and one brother was gouty. Sixteen years previous to admission the patient had had a vesical calculus removed, and a year later he had passed through a moderately severe typhoid fever. He was in the habit of drinking five or six pints of beer a day and two or three "rums" before breakfast. Five years ago he had had severe epistaxis. On January 1, after having been in bed for about an hour, he felt a gurgling sensation in his throat, and his mouth filled with something which, on striking a light, he was horrified to find was blood. He brought up over half a pint. The house officer diagnosed hæmatemesis and put him to bed in the ward at once. Seen by the physician next day, the patient was a big stout man with a florid complexion and multiple dilated venules on nose and cheeks. His sclerotics were slightly jaundiced. There was no anasarca or ascites, but the liver could be felt with ease, its edge coming two inches below the costal margin in the right nipple line and the surface seeming to be hard and smooth. The lungs were emphysematous. There were no signs of pulmonary tuberculosis. The pulse was 82, regular, and not of high tension. The radial artery was not thickened. The cardiac impulse could neither be seen nor felt. The area of cardiac dulness was diminished on account of the emphysema. A soft systolic apical bruit could be traced outwards for some little distance into the left axilla.

The urine was of specific gravity 1018, with just a trace of albumen present; there were no renal tube-casts. The man was coughing up bright-red frothy blood mixed with a little mucus. Hæmoptysis was now diagnosed. The previous error of diagnosing hæmatemesis depended on the alcoholic history, the alcoholic appearance of the patient, the enlargement, or apparent enlargement, of his liver; on careful observation it became clear that the blood was coughed up and not vomited; it was also mixed with definite sputum. The cause in this case was not tuberculosis but mitral disease.

